# Engineering Two-Dimensional Multilevel Supramolecular Assemblies from a Bifunctional Ligand on Au(111)

**DOI:** 10.3390/molecules28135116

**Published:** 2023-06-29

**Authors:** Rongyu Tang, Yang Song, Lizhi Zhang, Ziliang Shi

**Affiliations:** 1 Center for Soft Condensed Matter Physics and Interdisciplinary Research, School of Physical Science and Technology, Soochow University, Suzhou 215006, China; 20204208014@stu.suda.edu.cn; 2 Laboratory of Theoretical and Computational Nanoscience, National Center for Nanoscience and Technology, Beijing 100190, China; songy2022@nanoctr.cn

**Keywords:** two-dimensional supramolecular self-assembly, metal-organic coordination, surface, scanning tunneling microscopy, density functional theory

## Abstract

Herein, we demonstrate the supramolecular assemblies from a bifunctional ligand on Au(111), towards engineering two-dimensional (metal-) organic multilevel nanostructures. The bifunctional ligand employed, including two Br atoms and one carboxylic terminal, offers multiple bonding motifs with different configurations and binding energies. These bonding motifs are highly self-selective and self-recognizable, and thus afford the formation of subunits that contribute to engineering multilevel self-assemblies. Our scanning tunneling microscopy experiments, in combination with the density functional theory calculations, revealed various hydrogen, halogen and alkali-carboxylate bonding motifs dictating the different levels of the assemblies. The multilevel assembly protocol based on a judicious choice of multiple bonding motifs guarantees a deliberate control of surface-confined (metal-) organic nanostructures. Our findings may present new opportunities for the fabrication of complex two-dimensional (metal-) organic nanostructures with potential in applications of functionally diverse nanomaterials.

## 1. Introduction

On-surface supramolecular (metal-coordination) self-assembly based on noncovalent chemical interactions (e.g., hydrogen, halogen and metal-organic coordination bonding) has become an important strategy in the bottom-up engineering of low-dimensional nanostructures [1,2,3,4,5,6,7]. Because of the high selectivity, reversibility and directionality of noncovalent interactions, the judicious choice of multiple noncovalent bonding motifs allows one to deliberately control the thermodynamic and kinetic processes of a self-assembly and to create complex nanostructures, affording a variety of structural and chemical properties. For instance, the secondary building units (SBUs) approach has been developed to engineer a large number of three-dimensional porous metal–organic frameworks with various pores sizes, geometries and functionalities [8,9]. The strategy of hierarchical self-assembly, bestowing multiple noninterfering interactions involved, has succeeded in constructing complex multilevel nanoarchitectures [10,11,12].

Nevertheless, the use of multiple (or multifunctional) ligands brings out multiple bonding motifs, which often have different configurations and binding energies. The corresponding self-assembly may proceed with the competition and collaboration of distinct bonding motifs, which interfere in the assembly processes and thus result in unwanted by-products. The rational design of the target multilevel nanoarchitectures is a huge challenge. A comprehensive understanding of the thermodynamic and kinetic processes driven by the multiple bonding motifs involved at the single-molecule level is necessary.

Herein, we have investigated the two-dimensional multilevel assemblies out of the bifunctional organic ligand DBA (2, 5-dibromobenzoic acid), and the assemblies assisted with alkali halides on Au(111). The ligand has one carboxylic (COOH) and two Br terminals, which offer multiple bonding motifs to the corresponding assemblies, including COOH⋯COOH [13,14,15,16,17,18], Br⋯H hydrogen bonding [19,20,21,22], Br⋯Br halogen bonding [20,23] and alkali-COO ionic coordination bonding [24,25,26]. Based on the major bonding motifs, the resulting first-level subunits afford distinct configurations and functional terminals, which determine the topologies of the resultant multilevel nanoarchitectures. Scanning tunneling microscopy (STM) experiments, in combination with density functional theory (DFT) calculations, revealed that the emerging bonding motifs coexisted, competed and collaborated in the different levels of the self-assembled structures and finally generated various (metal-) organic nanostructures. Furthermore, by using two different alkali halides (NaCl and CsCl), we have explored the configurational adaptability [26,27,28] and the size effect of alkali coordination complexes [26,29,30,31], and have achieved a reversible structural conversion for the Cs-coordination assemblies. Our findings present new opportunities for the fabrication of complex two-dimensional (metal-) organic nanostructures with potential in applications of functionally diverse nanomaterials.

## 2. Results and Discussion

### 2.1. Self-Assembly of DBA on a Pristine Au(111)

**Figure 1:**
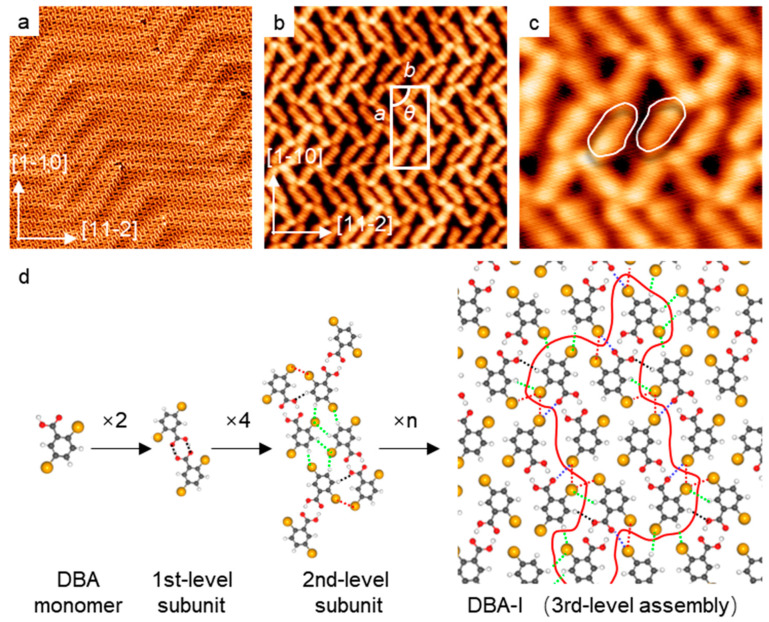
The self-assembled structure, namely, DBA-I, out of DBA on Au(111). (**a**) Large-scale STM image (50 nm × 50 nm; U = −1.2 V, I = 0.2 nA). (**b**) High-resolution STM image (10 nm × 10 nm; U = −1.2 V, I = 0.2 nA). The parameters of the unit cell: *a* = 3.29 nm, *b* = 1.45 nm and *θ* = 90°. (**c**) The topographic details of DBA monomers (2.5 nm × 2.5 nm; U = 1.2 V, I = 0.2 nA). (**d**) The multilevel assembly of DBA-I. The green, black, red and blue dotted lines represent Br⋯H, O⋯H, Br⋯Br and Br⋯O bonding motifs, respectively. The red outline denotes one 2nd-level subunit in the DBA-I assembly. Colors: Br, yellow; O, red; C, grey; H, white.

DBA molecules were self-assembled into a multilevel network structure, namely DBA-I, on a pristine Au(111) surface held at room temperature (RT)—see the STM overview in Figure 1a. The high-resolution STM topographic image (Figure 1b) depicts the unit cell with parameters: *a* = 3.29 nm, *b* = 1.45 nm and *θ* = 90°; the vector ***a*** is in alignment with the substrate vectors <1−10>. The individual molecule has two bright terminals representing Br atoms, while the carboxylic group appears in a lateral protrusion close to one of the Br terminals. Thus, each DBA is presented in an asymmetric shape; see the two outlined molecules in Figure 1c. Due to the relatively low catalytic activity of the Au(111) surface [32], DBA presumably maintained intact at this experimental condition. Accordingly, we have proposed a tentative structural model for DBA-I, where a three-level assembly scheme is presented; see Figure 1d. The first level of the assembly includes the formation of molecular dimers, where each two DBA monomers are interconnected through the two carboxylic groups. The resulting O-H⋯O distance in the dimeric unit measures 2.7 Å, falling in the range of the typical cyclic hydrogen bond [13,16]. The DBA dimers, with two different prochiral configurations, constitute the first-level subunits. Towards the second-level assembly, the four dimers are interconnected mainly through complementary Br⋯H-C hydrogen bonds (green dotted lines), measuring a Br⋯H distance of 2.5–3.1 Å [19,20,22]. Our tentative model also suggests Br⋯Br (red dotted lines) and O⋯H (black dotted lines) bonds joining the second-level subunit (see the summary of complementary bonding motifs in Table S1 in Supporting Information (SI)). Finally, these second-level subunits (i.e., the aggregates of four dimers) are further interconnected mainly by the outmost Br terminals, which constitute complementary bonding motifs, including Br⋯H-C, Br⋯Br, Br⋯O (blue dotted lines) and O⋯H (black dotted lines). The resulting extended two-dimensional supramolecular assembly constitutes the third-level superstructure. In short, the COOH⋯COOH, Br⋯H-C (or O⋯H) and Br⋯Br bonding motifs coexist on surface, which, however, emerge in different structural subunits and afford a three-level supramolecular assembly. Note that we have assigned the dimeric unit assisted by COOH⋯COOH as the first-level subunit, because the bonding motif is of high directionality and orthogonality, and solely emerges in dimeric units. Distinct from COOH⋯COOH, the configuration of Br⋯Br, O⋯H and Br⋯H-C bonding is highly adaptable, and thus they are assigned as complementary bonding motifs in the high-level assemblies.

### 2.2. Self-Assembly of DBA in the Presence of NaCl

**Figure 2:**
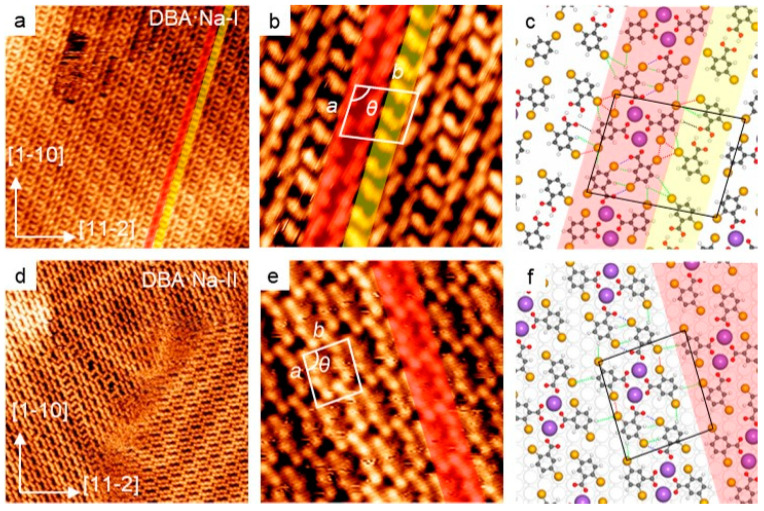
Self-assemblies of DBA in the presence of NaCl on Au(111). (**a**) An STM overview of DBA·Na-I (30 nm × 30 nm; U = −0.5 V, I = 0.5 nA). Row-I and -II are highlighted in yellow and red, respectively. (**b**) High-resolution STM image (8 nm × 8 nm; U = −0.5 V, I = 0.5 nA) showing the unit cell: *a* = 2.18 nm, *b* = 1.77 nm and *θ* = 97°. (**c**) The tentative structural model of DBA·Na-I. (**d**,**e**) STM overview (30 nm × 30 nm; U = −0.9 V, I = 0.2 nA) and high-resolution (8 nm × 8 nm; U = −0.5 V, I = 0.05 nA) images of DBA·Na-II. The parameters of the unit cell: *a* = 1.77 nm, *b* = 1.50 nm and *θ* = 94°. One row of 4DBA-2Na subunits is highlighted in red. (**f**) The DFT calculated structural model of DBA·Na-II. The green, red and blue dotted lines in (**c**,**f**) represent Br⋯H, Br⋯-Br and Br⋯O bonding motifs, respectively. Colors: Na, purple; Br, yellow; O, red; C, grey; H, white.

By customizing the configuration of the first-level subunit, one can design an alternative multilevel superstructure. To this end, we have co-deposited DBA and NaCl onto a Au(111) substrate held at RT, aiming to investigate the effect of alkali coordination in the assembly. An STM overview (Figure 2a) reveals a new structure (namely, DBA·Na-I) consisting of two types of rows; see the yellow (row-I) and red (row-II) shaded ribbons. Both rows propagate along the direction at ±14° from substrate vectors <11−2>. The high-resolution STM image (Figure 2b) reveals the unit cell with the parameters: *a* = 2.18 nm, *b* = 1.77 nm and *θ* = 97°. As shown in the tentative structural model (Figure 2c), in row-I are the DBA dimeric subunits from the two intact DBA molecules paired by cyclic COOH⋯COOH H-bonds with the measured bond length of 2.7 Å, which resembles that for DBA-I. We noted that no protrusions representing Na atoms were resolved in our STM observation. Nevertheless, considering that the structure only emerged after the co-deposition of NaCl, we have proposed that Na atoms exist in DBA·Na-I, particularly in row-II. Previous reports documented that Na was invisible in STM images, presumably because of its electronic state [26,28,30]. In addition, we propose that the four DBAs are deprotonated because of the bonding with Na [25,33]. In row-II, the two Na atoms 4.4 Å apart are embraced by four adjacent DBA molecules. The surrounding O atoms are about 2.2–2.3 Å away from the Na atom. This structural model is also supported by our DFT simulation (vide infra). The 4DBA-2Na unit forms a subunit directed mainly by Na⋯COO ionic bonding motifs. In addition, two Br⋯Br bonds (red dotted lines) assist the stabilization of a subunit. Note that the charges of alkali ions and carboxylate groups are ignored, since the substrate may partially screen the adsorbates charges. In between these subunits, the Br⋯H-C and Br⋯O bonding motifs (green and blue dotted lines) assist in constituting row-II. Finally, the two types of rows are mainly connected to each other by Br⋯Br and Br⋯H-C bonds (see the red and green dotted lines).

Intuitively, DBA·Na-I is a mixture of two assembled subunits; the dimers in row-I have appeared in DBA-I. Indeed, the pure phase (namely, DBA·Na-II) solely out of the row-II (highlighted in red) emerged after a thermal annealing at 406 K to the sample; see the STM overview shown in Figure 2d. Considering that DBA can be evaporated at RT, we have proposed that the 406 K annealing led to the desorption of DBA. The reduced DBA/Na ratio excluded row-I, which did not contain Na species. Now, the unit cell consists of one 4DBA-2Na subunit and (Figure 2e) measures *a* = 1.77 nm, *b* = 1.50 nm, and *θ* = 94°; ***a*** is in alignment with substrate vectors ±14° from <11−2> (as the same as DBA·Na-I). To interpret our STM results, we have carried out a DFT simulation according to our high-resolution STM observations. The geometric optimized atomic structural model (Figure 2f) gives a molecular supercell (√39 × √31)R95°, agreeing well with the experimental results. As indicated in Figure 2f, the Na pair acts as a dimetallic coordination center to link COO end groups belonging to the four nearest DBAs. These 4DBA-2Na complexes serve as the first-level subunits for the formation of DBA·Na-II, mainly via Br⋯H-C bonding motifs (green dotted lines), assisted simultaneously by the complementary Br⋯O halogen bonding (blue dotted lines); see details in Table S1 in the SI. Notably, the corresponding STM simulation (see Figure S1 in SI) produces depressions corresponding to the Na atoms, supporting our experimental observations. To summarize, by designing the first-level subunit through Na⋯COO bonding, we have achieved a two-level assembly of DBAs in the presence of NaCl, where Na⋯COO (and Br⋯Br) and Br⋯H-C (Br⋯O) bonding motifs emerge in different levels. In comparison with the first-level subunit for DBA-I, the 4DBA-2Na subunit has a distinct configuration and functional terminals, which, serving as the first-level subunit, determines the morphology of the final DBA·Na-II assemblies.

### 2.3. Size Effect of Alkali Coordination Centers

**Figure 3:**
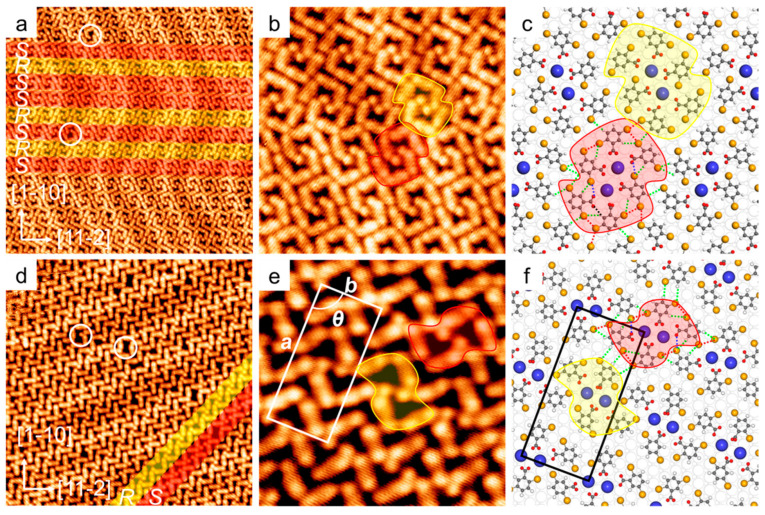
Self-assembled structure of DBA in the presence of CsCl on Au(111). (**a**) An STM overview of DBA·Cs-I (30 × 30 nm; U = −1.2 V, I = 0.05 nA). Rows-*R* and -*S* are highlighted in yellow and red, respectively. (**b**) High-resolution STM image (10 × 10 nm; U = −1.2 V, I = 0.05 nA). (**c**) The DFT calculated structure model of DBA·Cs-I. (**d**) The STM overview of DBA·Cs-II (20 nm × 20 nm; U = −1.0 V, I = 0.2 nA). (**e**) High-resolution STM (6 nm × 6 nm; U = −1.2 V, I = 0.1 nA). The parameters of the unit cell: *a* = 3.45 nm, *b* = 1.61 nm, *θ* = 90°. (**f**) The DFT calculated structure model for DBA·Cs-II. The red and green dotted lines in (**c**,**f**) represent Br⋯Br and Br⋯H bonds, respectively. Colors: Cs, blue; Br, yellow; O, red; C, grey; H, white.

To further explore the size effect of the alkali metals in the multilevel self-assembly, we have co-deposited DBA with CsCl onto a Au(111) substrate held at RT. Interestingly, there existed two assembled structures, namely, DBA·Cs-I and DBA·Cs-II (Figure 3), determined by the ratio of DBA to Cs (vide infra). Again, the two structures are presented in a three-level organization. In particular, as shown in Figure 3a, DBA·Cs-I consists of two types of rows, namely row-*R* (yellow) and row-*S* (red). Each row has the periodicity of 1.93 nm along the substrate vectors <11−2> and includes the subunits with a solely prochiral feature (*R* or *S*). Notably, both rows are stacked laterally in a random order, which leads to a non-periodic arrangement in the direction along the substrate vectors <1−10>. The missing dots (circles in Figure 3a) and a close look (Figure 3b) reveal that each two Cs atoms, appearing in bright protrusions, serve as a bimetallic coordination center and anchor six surrounding DBA molecules, forming a prochiral 6DBA-2Cs subunit; see the two frames in yellow and red. Accordingly, we have performed DFT simulation, which renders an optimized geometry for the atomic structural model of DBA·Cs-I structure; Figure 3c shows a two-dimensional periodic assembly for the purpose of calculation. The individual Cs atoms in each pair are 5.3 Å apart, and the distance from the surrounding O atoms is 3.2 Å. Such a resultant first-level subunit resembles that in DBA·Na-II, where Na pairs afford the coordination centers. However, because of the large vdW radii of Cs with respect to Na, a high coordination number is obtained. The first-level subunit, 6DBA-2Cs, is stabilized mainly by Cs⋯COO bonding and assisted by complementary Br⋯H-C and Br⋯Br bondings (green and red dotted lines). Since all COO terminals point towards the center within a subunit, only Br terminals (and peripheral H atoms) remain available for the high-level assembly. Thus, the second-level assembly, i.e., the row-*R*/-*S*, is established solely by Br⋯H bonding motifs. The two types of rows are alternatively stacked into the third-level assembly, where Br⋯H and Br⋯Br bonding motifs stabilize the stacking; see details in Table S1 in SI.

As shown in Figure 3d, DBA·Cs-II is also a multilevel structure, which, however, is based on a different first-level subunit. Several dots are also missing (circles in Figure 3d), which presumably indicates the absence of Cs ions. Again, two types of rows are visible, namely, row-*R* and -*S*, highlighted in yellow and red, respectively. Figure 3e depicts a unit cell measuring *a* = 3.45 nm, *b* = 1.61 nm, and *θ* = 90°, which contains two 4DBA-2Cs subunits with two distinct prochiral features (see the two frames in yellow and red). The geometric optimized atomic structural model provides a well-consistent molecular supercell (√151 × √31)-R90°; see the black frame in Figure 3f. The model presents the first-level subunit 4DBA-2Cs, where two Cs atoms (4.4 Å apart) are embraced by four DBAs through Cs-O bonds (~3.1 Å). The same prochiral subunits are further interconnected, mainly through complementary Br⋯H-C and Br⋯Br (green and red dotted lines) bonding, into the second-level assemblies, i.e., the rows-*R*/-*S*. The final (third-level) assemblies involve the stacking of the two rows, in which the complementary bonding is solely Br⋯H-C; see details in Table S1 in SI. In comparison with DBA·Na-II based on the 4DBA-2Na first-level subunit, the 4DBA-2Cs subunit in DBA·Cs-II has the same stoichiometric ratio but features deep indentations in its configuration. As shown in Figure 3e,f, such a configuration allows the Br atoms, belonging to the nearest neighboring subunits, to mutually interact and form more Br⋯Br bondings. The presence of the two different first-level subunits suggests a configurational adaptability for Cs⋯COO bonding motifs. It is the configurational adaptability that allows us to realize the structural conversion of the two Cs-coordination assisted assemblies. In comparison with the first-level subunit in DBA-I that is stabilized by COOH⋯COOH, the first-level subunits in DBA·Na-II and DBA·Cs-I(-II) are all interconnected mainly by alkali-carboxylate ionic bonding; they are further interconnected mainly by Br⋯H-C hydrogen bonding and assisted by Br⋯Br and/or Br⋯O halogen bonding to form metal-organic multilevel supramolecular assembled structures.

### 2.4. Reversible Structural Conversion of DBA·Cs-I and -II, and DFT Calculation

We have observed the structural conversion between DBA·Cs-I and -II occurring at room temperature. In a sequential deposition experiment (see Figure S2, in SI), we first deposited 3 min DBA and 5 min CsCl to obtain the pure assembly of DBA·Cs-I; a following deposition of 3 min CsCl gave DBA·Cs-II; after the deposition of 3 min more DBA, DBA·Cs-I reappeared. Apparently, the high configurational adaptability of alkali coordination allows us to modulate the structural conversion by adjusting the ratio of DBA and CsCl. Finally, we annealed the sample to 450 K, and DBA·Cs-II reappeared. Such a conversion achieved by thermal treatment can be attributed to the desorption of DBA molecules at elevated temperatures, which reduces the stoichiometric ratio of DBA molecules to Cs atoms.

To shed light on the mechanism of the structural conversion between DBA·Cs-I and -II, we have conducted DFT calculations to quantitatively examine the energy schemes of the two structures. Table 1 lists the calculation results, showing the binding energy for the unit cell of the two assemblies. We note that the average binding energy per Cs atom in DBA·Cs-I (2.82 eV) is larger than that in DBA·Cs-II (2.81 eV) by 0.01 eV, and the average binding energy per DBA in DBA·Cs-II (1.40 eV) is larger than that in DBA·Cs-I (0.94 eV) by 0.46 eV. This result is consistent with the experimental observations, in which DBA·Cs-I is energetically preferred when Cs is less (DBA:Cs > 3:1), while DBA·Cs-II is predominant when DBA is less (DBA:Cs < 2:1). Therefore, the assembly is determined by the less component, because the less component prefers to form a larger binding energy to save the total energy of the system. Additionally, this result also reveals the configurational adaptability of Cs-coordination bonding motifs.

## 3. Materials and Methods

**STM.** All experiments were carried out in an ultrahigh-vacuum scanning tunneling microscopy (UHV-STM) system (Aarhus 150, SPECS GmbH, Berlin, Germany) with a base pressure ~3.0 × 10^−10^ mbar. The Au(111) single crystalline substrate was cleaned by cycles of Ar^+^ ions sputtering and thermal annealing at 800 K. The organic molecule DBA (2, 5-Dibromobenzoic acid, Aladdin Chemistry, 96%) contained by a glass crucible was sublimed at room temperature. NaCl (Sigma-Aldrich, 99.999%) and CsCl (Sigma-Aldrich, ≥99.999%) were evaporated by using organic molecular beam epitaxy (OMBE, Dedocon GmbH) at 240 °C and 340 °C, respectively. All STM images were obtained at room temperature in the constant current mode, with the bias voltage applied to the sample. The STM image processing was performed using WSxM 5.0 software [34].

**First-Principles Calculations.** DFT calculations were implemented in the Vienna Ab-initio Simulation Package (VASP) [35,36,37] using projector-augmented wave (PAW) [38,39] pseudopotential combined with the Perdew–Burke–Ernzerhof (PBE) [40,41] functional. The Grimme-D3 method [42] was employed to describe the van der Waals interaction between molecules and metal substrate [43]. The energy cutoff for the plane-wave basis was set to 500 eV. All Au(111) substrates were modeled by three layered slabs, with the bottom layer fixed and two top layers relaxed. The thicknesses of the vacuum layers were all larger than 15 Å. The models of DBA·Na-II, DBA·Cs-I and DBA·Cs-II were built with (√39 × √31)-R95°, (4√3 × 14)-R90° and (√151 × √31)-R90° supercells of Au(111). The molecular layer and two upper metal layers were relaxed until the residual force on each of the relaxed atoms was less than 0.02 eV Å^−1^ and the break condition for the electronic self-consistent loop was 1 × 10^−6^ eV. The Brillouin zone was sampled by a (2 × 2 × 1) Gamma-centered k-mesh for DBA·Na-II and a (1 × 1 × 1) k-mesh for DBA·Cs-I and -II.

## 4. Conclusions

To conclude, through tuning multiple bonding motifs within the on-surface assemblies of the bifunctional ligand DBA, we have achieved five different types of multilevel assembled nanostructures (DBA-I, DBA·Na-I (-II) and DBA·Cs-I (-II)) with complex geometries on Au(111). The directionality of cyclic COOH⋯COOH bonding, the configurational adaptability, and the size effect of alkali coordination centers (Na/Cs⋯COO) are used to design the first-level subunits. Their peculiar geometries and terminal functions guide the following structural levels, yielding various multilevel nanostructures. The structural conversion of the Cs⋯COO-assisted assemblies was examined and attributed to the bonding competition between two species. Our findings provide insightful information regarding the fabrication of complex supramolecular nanoarchitectures at the single-molecule level.

## Figures and Tables

**Table 1:** Summary of the calculated binding energy for the assemblies of DBA·Cs-I and -II.

Structure	DBA·Cs-I	DBA·Cs-II
No. of DBA	12	8
No. of Cs	4	4
*E_b_* (eV) ^a^	11.30	11.23
*E_b_* per DBA (eV)	0.94	1.40
*E_b_* per Cs (eV)	2.82	2.81

^a^: *E_b_* is the total binding energy between DBA and Cs per unit cell.

## Data Availability

Not applicable.

## Supplementary Materials

The following supporting information can be downloaded at: https://www.mdpi.com/article/10.3390/molecules28135116/s1, Table S1: Summary of complementary bonding motifs; Figure S1: STM simulation of the structures DBA·Na-II, DBA·Cs-I and -II; Figure S2: The reversible structural conversion of DBA·Cs-I and -II. References [21,44,45,46] are cited in the supplementary materials.
