# Supplementary material for: Engineering Two-Dimensional Multilevel Supramolecular Assemblies from a Bifunctional Ligand on Au(111)
Source: Molecules. 2023 Jun 29;28(13):5116. doi: 10.3390/molecules28135116 (PMC10343213; doi:10.3390/molecules28135116)
Supplement: Supplementary file 1 [file molecules-28-05116-s001.zip › molecules-2467529-supplementary.pdf]

Supporting Information *for*

Engineering Two-Dimensional Multilevel Supramolecular Assemblies from a Bifunctional Ligand on Au(111)

Rongyu Tang [1], Yang Song [2], Lizhi Zhang [2,*] and Ziliang Shi [1,*]

[1] Center for Soft Condensed Matter Physics and Interdisciplinary Research, School of Physical Science and Technology, Soochow University, Suzhou 215006, China; 20204208014@stu.suda.edu.cn

[2] Laboratory of Theoretical and Computational Nanoscience, National Center for Nanoscience and Technology, Beijing 100190, China; songy2022@nanoctr.cn

* Correspondence: zhanglz@nanoctr.cn (L.Z.); phzshi@suda.edu.cn (Z.S.)

Table of Contents

1) Table S1: Summary of complementary bonding motifs; 2

2) Figure S1: STM simulation of the structures DBA·Na-II, DBA·Cs-I and -II; 2

3) Figure S2: The reversible structural conversion of DBA·Cs-I and -II. 3

1) Summary of complementary bonding motifs.

In Table S1, we have summarized the number of the complementary bonding motifs for DBA-I and the three alkali-coordination nanostructures (DBA·Na-II, DBA·Cs-I and -II), according to our analysis of the corresponding DFT optimized atomic structural models. The chemical interactions of Br···Br, Br···O, Br···H and O···H are determined according to previous literatures [21,44-46].

Table S1. Summary complementary bonding motifs.

Structure	DBA-I [a]		DBA·Na-II		DBA·Cs-I			DBA·Cs-II		
Level	2nd	3rd	1st	2nd	1st	2nd	3rd	1st	2nd	3rd
Br···H	6	8	0	6	7	3	2	2	4	2.5
O···H	2	2	0	0	1	0	0	0	0	0
Br···Br	2	6	2	0	4	0	1	2	2	0
Br···O	0	4	0	2	2	0	0	0	1	0

[a]: The bond lengths in DBA-I are measured based on the tentative structural model.

2) STM simulation of the structures DBA·Na-II, DBA·Cs-I and -II.

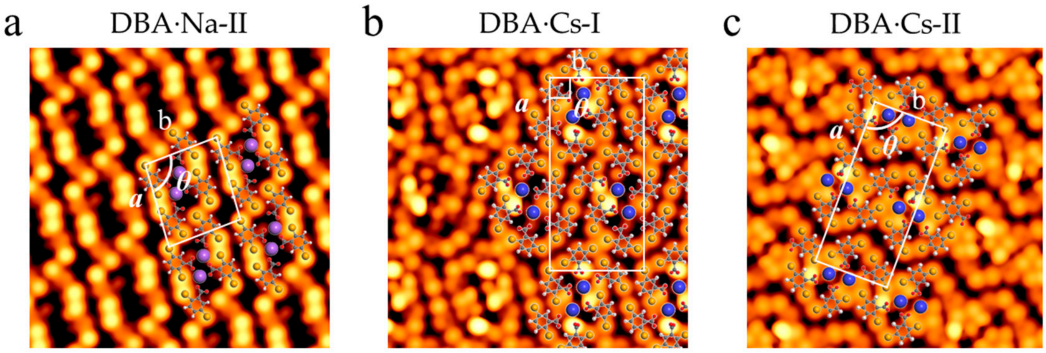

Figure S1. STM simulation of DBA·Na-II, DBA·Cs-I and DBA·Cs-II. Simulated STM images are covered with the corresponding atomic structural models. The white frames show the unit cells used in DFT calculations. The parameters of the unit cells (a, b and θ) are (a) 1.80 nm, 1.60 nm, 94°; (b) 4.04 nm, 2.00 nm, 90°; and (**c**) 3.54 nm, 1.61 nm, 90.4°. The energy range for STM simulations is from −1.0 eV to the Fermi level, which is comparable with the bias voltages in the STM experiments (−1.2 ~ −0.5 V).

3) The reversible structural conversion of DBA·Cs-I and -II.

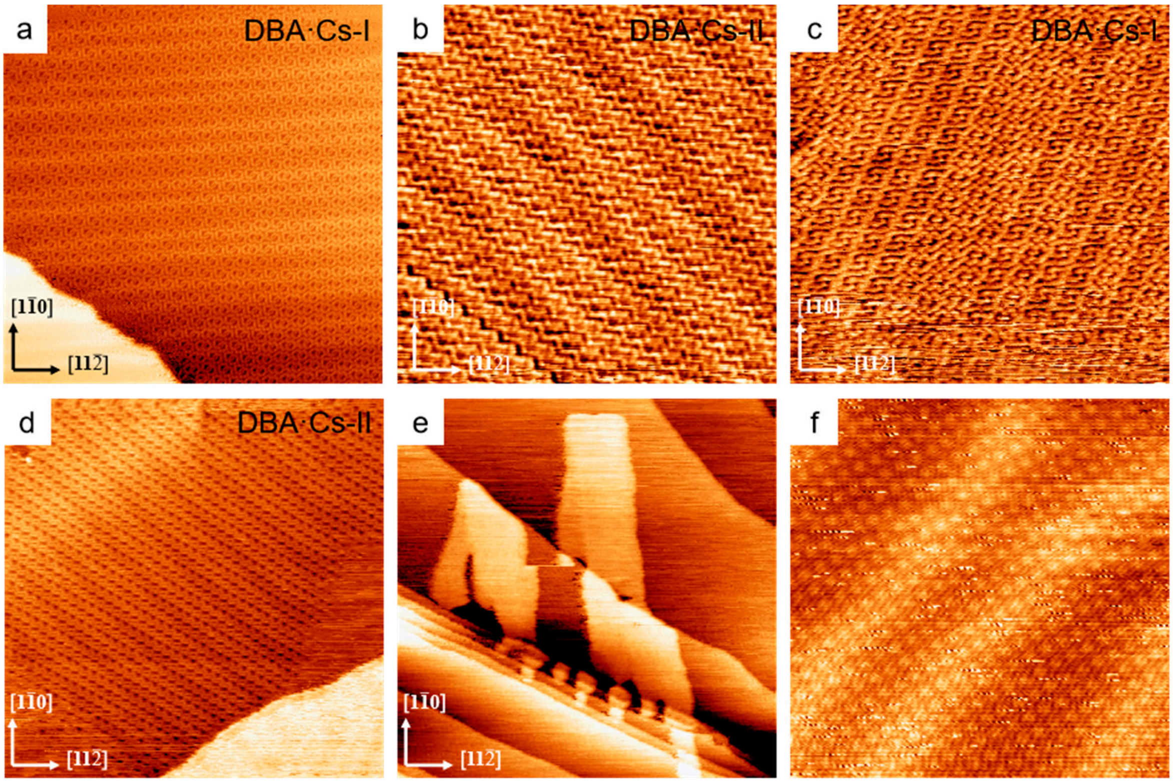

Figure S2. STM results of the reversible structural conversion of DBA·Cs-I and -II. (a) The pure phase of DBA·Cs-I after a codeposition of DBA (0.6 ML) with CsCl (0.16 ML) (50 × 50 nm; U = −1.2 V, I = 200 pA). (b) An STM image of the sample acquired after a post-deposition of CsCl (0.09 ML) (28 × 28nm; U = −1.2 V, I = 50 pA). (c) An STM overview of the sample after a post-deposition of DBA (0.3 ML) (30 × 30 nm; U = −2.6 V, I = 200 pA). (d) An STM overview of the sample after a 450 K-annealing (62 × 62 nm; U = −1.2 V, I = 50 pA). (e) A typical CsCl bilayer structure on Au(111) (300 × 300 nm; U = −0.6 V, I = 50 pA). (f) The atomic-resolution of the CsCl bilayer (10 × 10 nm; U = −1.6 V, I =50 pA), measuring the lattice constant of 4.8 Å. 1 ML of DBA refers to a full coverage of the DBA-I structure on the surface. 1 ML of CsCl refers to a full coverage of the CsCl bilayer on the surface.
